# Targeting programmed cell death ligand 1 by CRISPR/Cas9 in osteosarcoma cells

**DOI:** 10.18632/oncotarget.16326

**Published:** 2017-03-17

**Authors:** Yunfei Liao, Lulu Chen, Yong Feng, Jacson Shen, Yan Gao, Gregory Cote, Edwin Choy, David Harmon, Henry Mankin, Francis Hornicek, Zhenfeng Duan

**Affiliations:** ^1^ Department of Endocrinology, Wuhan Union Hospital, Tongji Medical College, Huazhong University of Science and Technology, Wuhan 430022, China; ^2^ Sarcoma Biology Laboratory, Department of Orthopaedic Surgery, Massachusetts General Hospital and Harvard Medical School, Boston 02114, Massachusetts, USA; ^3^ Department of Orthopaedic Surgery, Wuhan Union Hospital, Tongji Medical College, Huazhong University of Science and Technology, Wuhan 430022, China; ^4^ Division of Hematology and Oncology, Massachusetts General Hospital and Harvard Medical School, Boston 02114, Massachusetts, USA

**Keywords:** programmed cell death ligand 1, osteosarcoma, CRISPR/Cas9, metastasis

## Abstract

Programmed cell death ligand 1 (PD-L1) is a transmembrane protein that is expressed on tumor cells that suppresses the T cell-mediated immune response. Therapies targeting the PD-L1 pathway promote anti-tumor immunity and have shown promising results in some types of cancers. However, the functional and therapeutic roles of PD-L1 in osteosarcoma remain largely unknown. In this study, we found that PD-L1 protein was expressed in osteosarcoma cell lines and tissue microarray of patient tumors. Tissue microarray immunohistochemistry analysis showed that the overall and five-year survival rates of patients with high levels of PD-L1 expression were significantly shorter than patients with low levels. High levels of PD-L1 expression were also associated with metastasis in osteosarcoma patients. Furthermore, we applied the Clustered Regularly Interspaced Short Palindromic Repeats (CRISPR)/Cas9 system to target PD-L1 gene at the DNA level in osteosarcoma cell lines. We found that the expression of PD-L1 could be efficiently disrupted by CRISPR/Cas9 system and PD-L1 knockdown increased drug sensitivities for doxorubicin and paclitaxel. These results suggest that PD-L1 is an independent prognostic factor in osteosarcoma and that PD-L1 knockout by CRISPR/Cas9 may be a therapeutic approach for the treatment of osteosarcoma.

## INTRODUCTION

Osteosarcoma is the most common type of primary malignant bone tumor affecting both children and adults. Current treatment for osteosarcoma is effective but limited. While surgery alone can only result in long-term survival rates around 10–20%, surgery combined with chemotherapy has increased the disease-free survival rate to more than 60% [1]. However, despite aggressive chemotherapy, more than 30% of patients with localized osteosarcoma experience recurrent or progressive metastatic disease, and the average survival period after developing metastasis is less than one year [2]. Therefore, more effective therapeutic strategies are required for the treatment of osteosarcoma.

Programmed cell death ligand 1 (PD-L1) is the ligand for programmed cell death 1 (PD-1). As an immune inhibitory moiety, PD-L1 is expressed by most cell types including cancer cells. PD-L1 is able to deliver an inhibitory signal to PD-1 expressing T cells, resulting in immune system impairment [3]. Recent evidence strongly suggests that the activation of the PD-1/PD-L1 pathway represents a mechanism allowing tumors to escape the host's immune system [4, 5]. Therapies targeting this signaling pathway promote marked antitumor immunity and have shown promising results in a subset of solid tumors, including melanoma, lung cancer, and head and neck carcinomas [6]. PD-L1 protein is expressed in a wide range of human tumors [7]. In addition, studies on the relationship between PD-L1 expression and the disease outcomes have shown that PD-L1 expression significantly correlates with poor prognosis in kidney, ovarian, bladder, breast, liver, gastric, and pancreatic cancer [8]. Blocking the PD-1/PD-L1 pathway induced objective response rates of 6% to 17% and prolonged stabilization of the disease in patients with non-small-cell lung cancer, melanoma, or renal-cell cancer [9]. Moreover, immunohistochemical assessment of PD-L1 in pretreatment cancer specimens from 42 patients revealed that response to treatment was observed exclusively in PD-L1 positive tumors (9/25, 36%) [9]. Although the PD-1/PD-L1 pathway is heavily targeted for anticancer drug discovery, the functional and therapeutic roles of PD-L1 in osteosarcoma remain largely unknown. Our previous study of PD-L1 RNA expression by quantitative real-time RT-PCR showed that PD-L1 was expressed in over 80% of osteosarcoma patient samples [10].

PD-1 and PD-L1 based cancer immunotherapies normally require continuous treatment with anti-PD-1 or anti-PD-L1 antibody, which may be costly. Alternative therapeutic strategies of targeting PD-1 or PD-L1 are needed. The clustered regulatory interspaced short palindromic repeat (CRISPR)/Cas9-based RNA-guided DNA endonuclease technology has been widely adopted for its affordability, versatility, ease of use, and low cost [11, 12]. CRISPR/Cas9 provides a robust and highly efficient novel genome editing tool, which enables precise manipulation of specific genomic loci, and facilitates elucidation of target gene functions or diseases [13]. This tool has successfully been applied to induce manipulation of induced pluripotent stem cells (iPS), genome editing, and gene therapy studies [14–16]. The clinical trial of T-cell modulation by CRISPR/Cas9 in cancer immunotherapy will be performed in 2017 [17].

In this study, we detected the expressions of PD-L1 protein in osteosarcoma cell lines and patient specimens. We also evaluated the association between PD-L1 expression and clinical characteristics in osteosarcoma patients. The efficiency of a CRISPR/Cas9 system on disruption of PD-L1 expression was assessed in osteosarcoma cells. Furthermore, the effects of PD-L1 knockout on osteosarcoma cell growth, migration, invasion, and drug resistance to doxorubicin and paclitaxel were determined.

## RESULTS

### Identification of PD-L1 expression in osteosarcoma cell lines and patients

**Figure 1:**
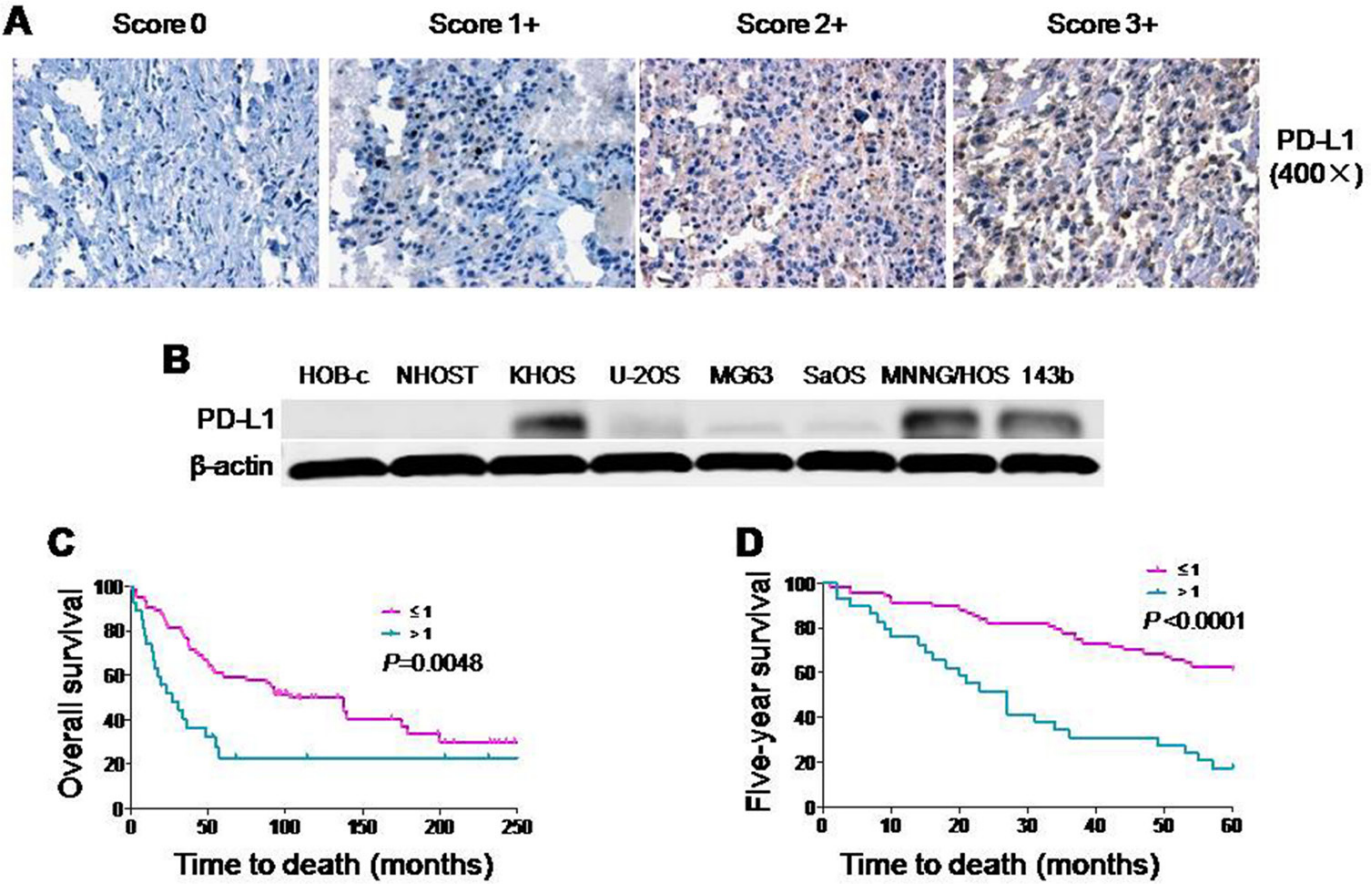
Expression of PD-L1 protein in osteosarcoma cell lines and osteosarcoma patient tissues (**A**) Representative images of different immunohistochemical staining intensities of PD-L1 are shown in osteosarcoma tissues. The percentage of cells showing positive cytoplasmic staining for PD-L1 was calculated by reviewing the entire spot. Based on the PD-L1 staining intensities in the tumor samples, the staining patterns were categorized into 4 groups: no staining (0), weak staining (1+), moderate staining (2+) and intense staining (3+) (Original magnification, 400×). (**B**) Expression of PD-L1 protein in osteosarcoma cell lines and normal osteoblast cell lines. (**C**) Kaplan-Meier overall survival curve of patients with osteosarcoma were subgrouped as either PD-L1 low staining (staining ≤ 1) or high staining (staining ≥ 2). (**D**) Kaplan-Meier five-year survival curve of patients with osteosarcoma were subgrouped as either PD-L1 low staining (staining ≤ 1) or high staining (staining ≥ 2).

PD-L1 expression has been demonstrated to play a crucial role in some tumor cell metastasis [18, 19]. However, PD-L1 expression in osteosarcoma remains unclear. In this study, we examined PD-L1 expression in a tissue microarray of patient tumors. Among the available specimens, 97 of 114 (85.1%) exhibited PD-L1 immunostaining in the cytoplasm, ranging from no staining (15 of 97, 15.5%), 1+ staining (43 of 97, 44.3%), 2+ staining (18 of 97, 18.6%), and 3+ staining (21 of 97, 21.6%); 17 specimens were not counted due to tissue loss on the TMA slide. Based on PD-L1 staining intensities in tumor samples, no staining (0) and weak staining (1+) specimens were classified as PD-L1-low (59.8%); moderate staining (2+) and intense staining (3+) were classified as PD-L1-high (40.2%) (Figure 1A). We observed that patients with metastasis have higher expressions of PD-L1 than non-metastatic patients. We next examined six osteosarcoma cell lines by Western blotting. PD-L1 was detected in most of the osteosarcoma cell lines. Among those osteosarcoma cell lines, MNNG/HOS and 143B, which are highly tumorigenic and metastatic, displayed the most abundant expression of PD-L1. In normal human osteoblast cell lines (HOB-c and NHOST), PD-L1 expression was extremely low and almost undetectable (Figure 1B). Therefore, PD-L1 protein is widely expressed and may be associated with metastasis both in osteosarcoma cell lines and in osteosarcoma patients.

### Correlation between PD-L1 and clinical characteristics in osteosarcoma patients

There were significant differences in PD-L1 expression in patient samples comparing absent metastasis and present metastasis (*P* < 0.001). In addition, patients with high expression of PD-L1 had a trend of poor response to preoperative chemotherapy (*P* = 0.1642). However, there were no significant relationship between PD-L1 expression and the other clinic pathological features of the human tumor samples, such as age, gender, or the recurrence (Table 1). Kaplan-Meier analysis showed that osteosarcoma patients in the high PD-L1 expression group had a lower overall survival rate compared with patients in the low PD-L1 expression group (*P* = 0.0048) (Figure 1C). Meanwhile, compared with low expression of PD-L1, patients with high expression of PD-L1 possessed a worse five-year survival rate (*P* < 0.001). Univariate Cox regression analysis indicated that PD-L1 expression was the independent prognostic factor of overall and five-year survival rates (*P* = 0.045 and 0.009) (Supplementary Table 1). Taking these data together, we found that there was a close relationship between PD-L1 expression and clinic pathological features (especially metastasis) of osteosarcoma.

**Table 1:** The relationship between PD-L1 expression and clinicopathological features of osteosarcoma

	Clinic pathological features	No. of cases (%)	PD-L1 expression High (%)	PD-L1 expression Low (%)	*P* value
All patients		72 (100)			
Age, y	≤ 18	32 (52.2)	15 (51.4)	17 (48.6)	0.4763
	> 18	40 (47.8)	15 (31.3)	25(68.7)	
Gender	Male	43 (62.1)	17 (40.0)	26 (60.0)	0.8079
	Female	29 (37.9)	13 (44.4)	16 (55.6)	
Metastasis	Absent	24 (32.8)	2 (40.9)	22 (59.1)	< 0.0001
	Present	48 (67.2)	28 (42.2)	20 (57.8)	
Recurrence	Absent	49 (70.1)	19 (42.6)	30 (57.4)	0.6089
	Present	23 (29.9)	11 (40.0)	12 (60.0)	
Response to preoperative chemotherapy	Good*	10 (12.1)	3 (62.5)	7 (37.5)	0.1642
	Poor	40 (54.6)	23 (41.7)	17 (58.3)	
	N/A	22 (33.3)	4 (59.1)	18 (40.9)	
PD-L1 expression	High		30 (41.8)		
	Low			42 (58.2)	

* Necrosis over 90%.

### Construction and verification of PD-L1 CRISPR/Cas9 *in vitro*

**Figure 2:**
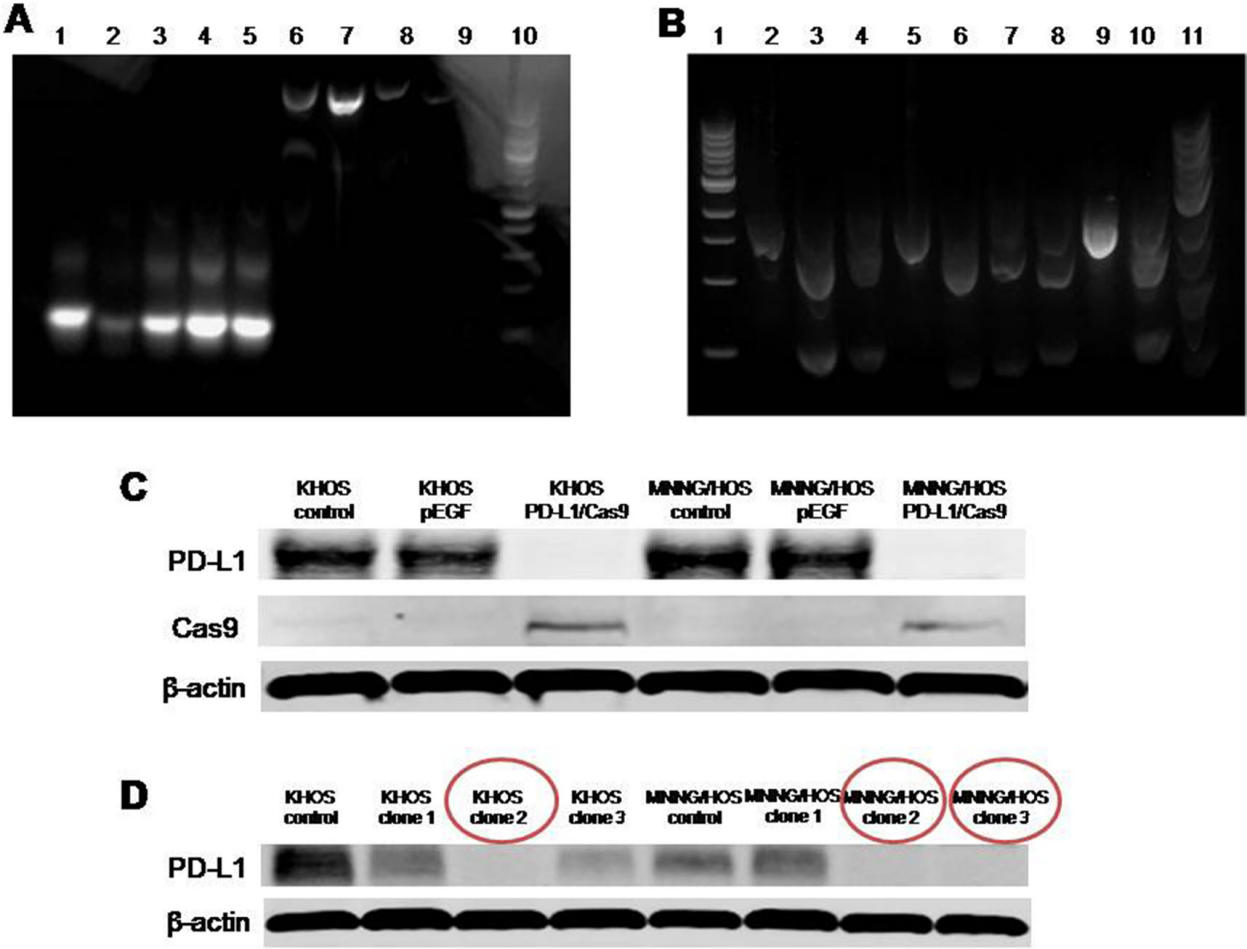
Verification of PD-L1 CRISPR/Cas9 *in vitro* (**A**) All of the five designed sgRNAs were generated by performing PCR with the included Guide-it Scaffold Template and the designed primer. The template was then transcribed with the included Guide-it T7 Polymerase Mix to create a sgRNA containing the target PD-L1 sequence. All five of the designed sgRNAs showed a 140bp PCR product. Lane 1, PD-L1 sgRNA-1; Lane 2, PD-L1 sgRNA-2; Lane 3, PD-L1 sgRNA-3; Lane 4, PD-L1 sgRNA-4; Lane 5, PD-L1 sgRNA-5; Lane 6, PD-L1 A high concentration; Lane 7, PD-L1 B high concentration; Lane 8, PD-L1 A low concentration; Lane 9, PD-L1 B low concentration; Lane 10, Ladder. (**B**) A cleavage reaction on this template was then performed by using the purified sgRNA in combination with the included Guide-it Recombinant Cas9 Nuclease. Each of the five sgRNA plus Cas9 could cut the specific DNA sequence from PD-L1 into two parts. Lane 1, Ladder only; Lane 2, PD-L1 A; Lane 3, PD-L1 sgRNA-1/ PD-L1 A; Lane 4, PD-L1 sgRNA-2/ PD-L1 A; Lane 5, PD-L1 B; Lane 6, PD-L1 sgRNA-3/PD-L1 B; Lane 7, PD-L1 sgRNA-4/PD-L1 B; Lane 8, PD-L1 sgRNA-5/PD-L1 B; Lane 9, Control template; Lane 10, Positive control; Lane11, Ladder mixed with loading buffer (may reduce smear). (**C**) Expression of PD-L1 protein in osteosarcoma cells transfected with PD-L1 CRISPR/Cas9. (**D**) Expression of PD-L1 protein in osteosarcoma cells on 4th passage after fluorescence activated cell sorting (FACS) sorting.

Before the transfection of plasmid with different sgRNA into osteosarcoma cells, verification of PD-L1 CRISPR/Cas9 *in vitro* was performed. A sgRNA consists of a crRNA sequence that binds to a specific DNA target, and a tracrRNA sequence that binds to Cas9 protein. When a sgRNA binds to a recombinant form of the Cas9 protein that has double-stranded DNA endonuclease activity, the resulting complex will produce target-specific double-stranded cleavage. Cellular repair, which is error-prone, will take place at the cleavage site, and may result in a mutation that can knock out a gene. In Figure 2A, all of the five designed sgRNAs showed a 140bp PCR product as expect. In Figure 2B, similar to the positive control, all five of the sgRNA plus Cas9 could cut the specific DNA sequence from PD-L1 into two parts. In Figure 2C, the PD-L1 expression was knocked out both in KHOS-PD-L1-Cas9 and in MNNG/HOS-PD-L1-Cas9 cells, while there were no changes in PD-L1 expression in KHOS-pEGFP and MNNG/HOS-pEGFP cells. These data demonstrated that each of the PD-L1 CRISPR/Cas9 constructs could effectively target the PD-L1 gene.

### Generation of osteosarcoma cell lines with constitutive knockout of PD-L1 expression

**Figure 3:**
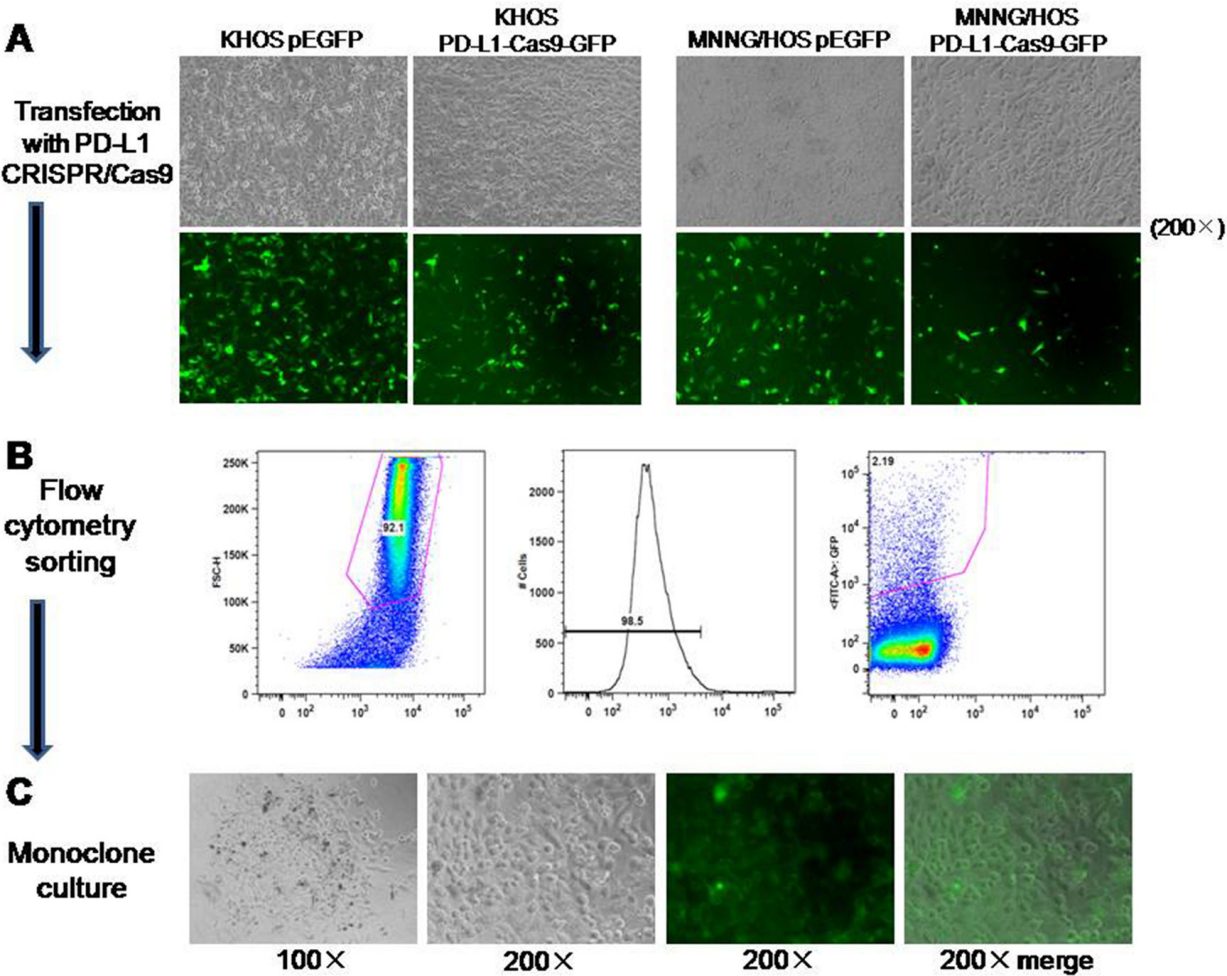
Generation of osteosarcoma cell lines with constitutive knockout of PD-L1 expression (**A**) Transfection of osteosarcoma cells (KHOS and MNNG/HOS) with PD-L1 CRISPR/Cas9 plasmid and GFP resulted in approximately 50–75% positive cells as observed by green fluorescence. (**B**) FACS was performed based on GFP expression. (**C**) Monoclone was picked up according to the GFP expression from 96-well.

According to the results of *in vitro* verification, we chose two different sgRNAs (#2 and #3) individually targeting at exon 2 and 3 of PD-L1 gene for the generation of osteosarcoma cell lines with constitutive knockout of PD-L1 expression. Transfection of osteosarcoma cells (KHOS and MNNG/HOS) with PD-L1 CRISPR/Cas9 plasmid and GFP resulted in transfection of approximately 50–75% of the cells as observed by green fluorescence (Figure 3A). Subsequently, FACS cell sorting was performed based on GFP expression (Figure 3B) and enabled enrichment of PD-L1 knock out cells (Figure 3C). The effectiveness of PD-L1 CRISPR/Cas9 was evaluated by the expression of PD-L1 protein. After four passages, three out of six clones generated from the FACS sorted and cultured cells showed complete loss of PD-L1 expression (KHOS clone #2, MNNG/HOS clone #2, and MNNG/HOS clone #3). In Figure 2D, KHOS clone #1 and #2 show partial loss of PD-L1 expression, and MNNG/HOS clone #1 shows no effect on PD-L1 expression. This maintenance of significant inhibition of PD-L1 expression leads us to consider KHOS clone #2, MNNG/HOS clone #2, and MNNG/HOS clone #3 as the atypical knockout that precluded further characterization.

### Knockout of PD-L1 expression by PD-L1 CRISPR/Cas9 inhibits osteosarcoma cell drug resistance to doxorubicin and paclitaxel

**Figure 4:**
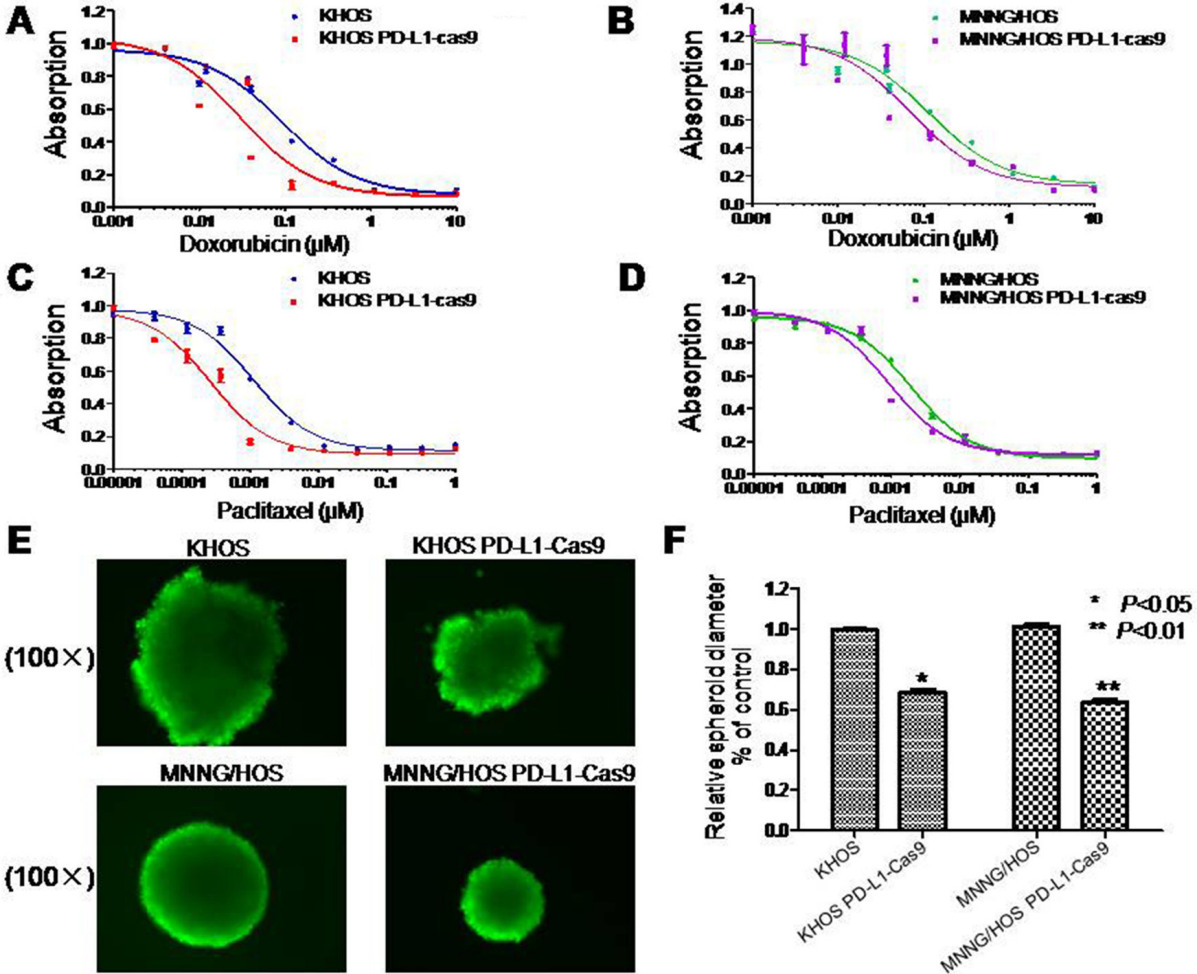
Drug resistance and three dimensional culture in osteosarcoma cell lines with and without PD-L1 CRISPR/Cas9 (**A**, **B**) Effects of PD-L1 on osteosarcoma drug resistance to doxorubicin and paclitaxel were assessed by MTT assay. Cells were dosed with doxorubicin in increasing concentrations up to 10 μM. (**C**, **D**) Cells were dosed with paclitaxel in increasing concentrations up to 1 μM. (**E**, **F**) PD-L1 CRISPR/Cas9 suppressed sphere formation both in KHOS PD-L1 CRISPR/Cas9 and MNNG/HOS PD-L1 CRISPR/Cas9 cells in three-dimensional culture. The assay was conducted in duplicate. *P < 0.05, **P < 0.01 (compared with control cells).

Doxorubicin and paclitaxel are commonly used in the treatment of osteosarcoma. However, there are many osteosarcoma patients resistant to doxorubicin and paclitaxel chemotherapy. In this study, the MTT assay was applied to evaluate the role of PD-L1 in the osteosarcoma cell drug resistance to doxorubicin and paclitaxel. The IC50 of doxorubicin in KHOS PD-L1-Cas9 was 0.030 μM and the IC50 of doxorubicin in KHOS was 0.092 μM (Figure 4A). Meanwhile, the IC50 of doxorubicin in MNNG/HOS PD-L1-Cas9 was 0.0705 μM and the IC50 of doxorubicin in MNNG/HOS was 0.1152 μM (Figure 4B). The results of paclitaxel in osteosarcoma cells remain the same. The IC50 of paclitaxel in KHOS PD-L1-Cas9 was 0.00026 μM and the IC50 in KHOS was 0.00113 μM (Figure 4C). The IC50 of paclitaxel in MNNG/HOS PD-L1-Cas9 was 0.00092 μM and the IC50 in MNNG/HOS was 0.0020 μM (Figure 4D). The results of the MTT assay demonstrated that PD-L1 may take part in the drug resistance of osteosarcoma and might be a potential target in clinical treatment.

### Knockout of PD-L1 expression by CRISPR/Cas9 inhibits osteosarcoma cell spheroid formation in 3D culture

In order to mimic the *in vivo* environment and detect the effect of PD-L1 on osteosarcoma tumorigenicity, a 3D cell culture was performed. Osteosarcoma cells without treatments were considered as control. After seven days of culture in 3D, the diameter of KHOS spheroids formed by PD-L1 CRISPR/Cas9 intervention was 68% of control cells, which was relatively smaller than blank controls (*P* = 0.008). Similar results were seen in MNNG/HOS cells. The diameter of MNNG/HOS spheroids formed by PD-L1 CRISPR/Cas9 intervention was 64% of control cells (*P* = 0.012) (Figure 4E and 4F). Thereby, the results suggest that PD-L1 CRISPR/Cas9 significantly depressed the growth and tumorigenicity of osteosarcoma cells.

### Knockout of PD-L1 expression by PD-L1 CRISPR/Cas9 showed no significant effects on osteosarcoma cell migration and invasion

**Figure 5:**
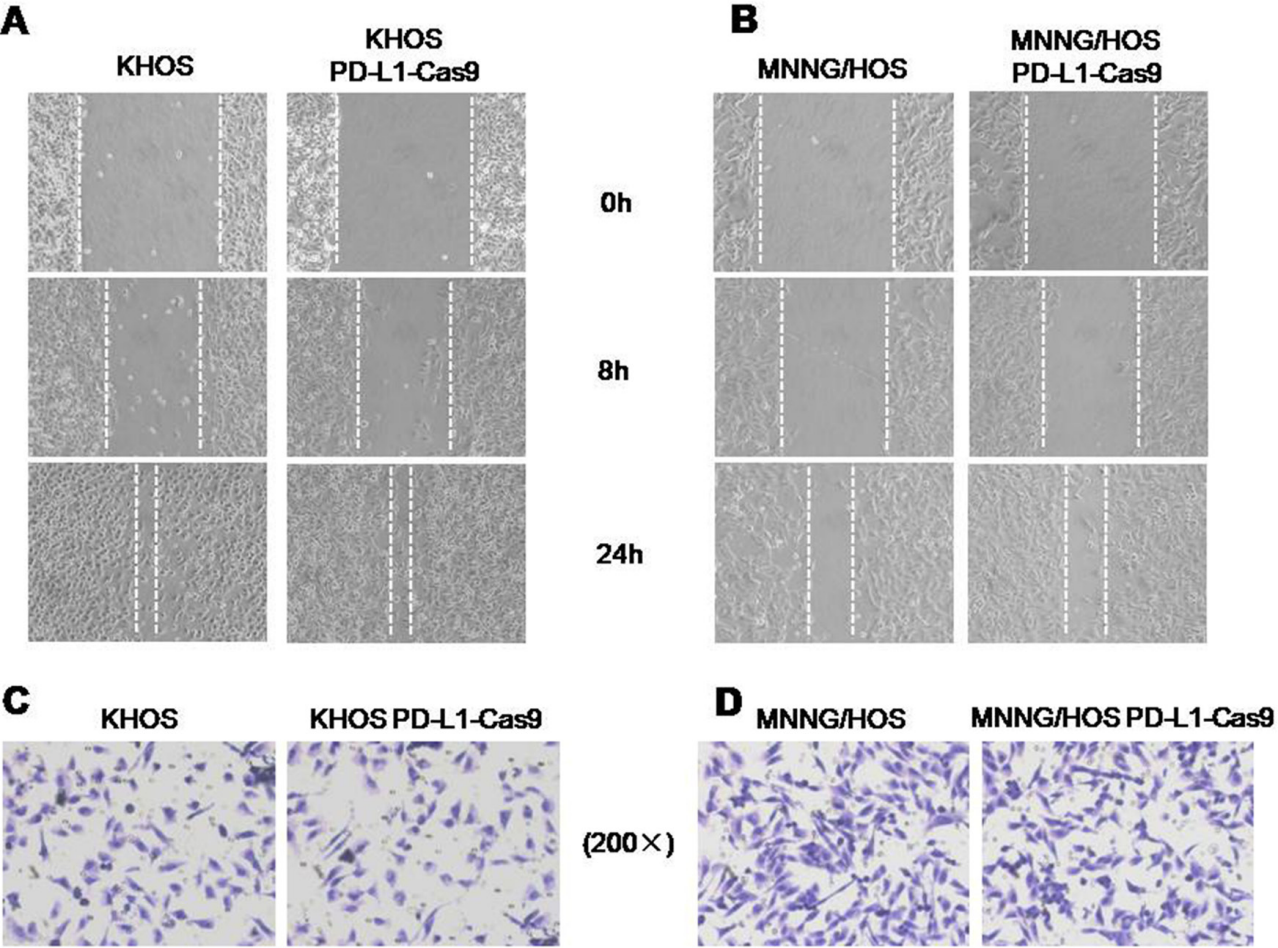
The migratory and invasive activities in osteosarcoma cell lines with and without PD-L1 CRISPR/Cas9 (**A**) Micrographs of osteosarcoma cells at 0, 8, and 24 hours after wounding. (**B**) Migration distance of KHOS and KHOS PD-L1 CRISPR/Cas9 for each time point and condition. (**C**) Migration distance of MNNG/HOS and MNNG/HOSPD-L1 CRISPR/Cas9 for each time point and condition. (**D**) Micrographs of osteosarcoma cells with and without PD-L1 CRISPR/Cas9. The invading cells were stained with hematoxylin. The average numbers of invasive osteosarcoma cells with and without PD-L1 CRISPR/Cas9. *P < 0.05, **P < 0.01 (comparison of transfected cells with control cells using Student's *t*-test).

Migration and invasion capabilities are crucial prerequisites for metastatic osteosarcoma. In this study, we evaluated whether knockout of PD-L1 expression could affect the migratory and invasive activities of osteosarcoma cells by the wound healing assay and Matrigel invasion assay. In the wound healing assay, wounds were almost recovered after 24 hours of migration both in blank control cells and KHOS PD-L1-Cas9 and MNNG/HOS PD-L1-Cas9 cells (Figure 5A). In Matrigel invasion assays, the average number of osteosarcoma cells that invaded through the Matrigel showed no significant differences between CRISPR/Cas9 cells and blank control cells. However, there was a trend that the numbers of invading cells of KHOS PD-L1-Cas9 and MNNG/HOS PD-L1-Cas9 decreased compared with KHOS and MNNG/HOS cells (Figure 5D). These data suggest that migration and invasion might not be mediated by PD-L1 in osteosarcoma.

## DISCUSSION

In this study, we successfully established a PD-L1 CRISPR/Cas9 system and generated osteosarcoma cell lines with constitutive knockout of PD-L1 expression. By CRISPR/Cas9 system, PD-L1 has been verified to mediate osteosarcoma cell, growth and drug resistance to doxorubicin and paclitaxel. In addition, PD-L1 expression was also confirmed to be associated with metastasis in osteosarcoma and can be considered as a predictor for five-year and overall survival in osteosarcoma patients. This is the first report demonstrating the PD-L1 CRISPR/Cas9 system in osteosarcoma, which offers a new treatment approach for PD-L1 in osteosarcoma.

It is well-known that the immune regulatory receptor PD-1 and its ligand PD-L1 are important mechanisms of tumor immune tolerance and escape. PD-1 is a cell-surface receptor expressed on subsets of T and B lymphocytes, as well as on other immune cells. PD-L1 is a transmembrane protein expressed on antigen present cells (APC), such as on various cancer cells. The PD-1/PD-L1 complex transmits an inhibitory signal, which reduces the proliferation of CD8^+^ T cells at the lymph nodes and suppresses the immune response [20]. Previously, we demonstrated that high levels of PD-L1 mRNA are expressed in osteosarcoma, and that PD-L1 mRNA expression is positively correlated with tumor-infiltrating T-lymphocytes (TILs), which is regarded as a marker of metastasis [10]. In chordoma, the expression of PD-L1 protein has also been found to be associated with the presence of elevated TILs [21]. However, the role of PD-L1 protein directly in biological functions of osteosarcoma cell lines remains unclear. This study provides new evidence for the role of PD-L1 in osteosarcoma. We evaluated different osteosarcoma cell lines and osteoblast cell lines. Both the cell line and patient data demonstrate that PD-L1 may be involved in osteosarcoma biological features, especially metastasis. We observed by Western blot that PD-L1 was widely expressed in osteosarcoma cell lines as compared with osteoblast cell lines. Furthermore, the richest expression of PD-L1 was shown in the osteosarcoma cell lines with the highest tumorigenesis and metastatic potential (MNNG/HOS and 143B). Correspondingly, PD-L1 protein expression exhibited predominantly cytoplasmic staining and was detected in most patient specimens of the TMA assay. Moreover, there was a close relationship between PD-L1 expression and metastasis. The expression of PD-L1 protein was even an independent risk factor of metastasis in osteosarcoma patients. This was similar to studies on oral squamous cell carcinoma. Those patients with distant metastasis had high expression of PD-L1, which was analyzed by immunohistochemistry [18]. Another study with 107 cases of human gastric carcinoma suggested that high expression of PD-L1 was a risk factor and a new biomarker to predict the prognosis of gastric cancer. In their study, PD-L1 expression was significantly associated with depth of invasion, lymph node metastasis, pathological type, and overall survival [19]. Thus, these data contain important implications for the application of PD-L1 therapies in osteosarcoma treatment, especially in patients with metastasis.

The immune therapeutics targeting the PD-1/PD-L1 axis in cancer patients with a wide variety of malignancies is rapidly evolving with successful clinical studies [22]. Recently, the FDA approved the PD-1 antibodies, pembrolizumab and nivolumab, for the treatment of advanced melanoma and non-small cell lung cancer [23, 24]. Due to the uncertainty of clinical effectiveness and expensive cost of PD-L1 antibodies, finding other effective therapeutic strategies to target PD-L1 is still necessary. CRISPR/Cas9 is a very useful genomic editing tool and has been successfully applied to the treatment of some diseases. The CRISPR/Cas9 system is a prokaryotic immune system that confers resistance to foreign genetic elements such as plasmids and phages, and provides a form of acquired immunity. CRISPR spacers recognize and cut these exogenous genetic elements in a manner analogous to RNA interference in eukaryotic organisms. As it accurately targets DNA and is a genetically heritable modification, the CRISPR/Cas9 system has been widely used for gene editing (adding, disrupting, or changing the sequence of specific genes) and gene regulation in species throughout life [11, 12]. Furthermore, CRISPR/Cas9 offers great promise for targeting different stages of the viral life cycle, and has the capacity for mediating an effective and sustained genetic therapy against HBV and HIV [25, 26]. Moreover, CRISPR/Cas9-mediated knockout of some key oncogenes has been demonstrated to decrease the malignant potential of prostate cancer cells, and inhibit tumor growth and pulmonary metastasis in triple-negative breast cancer [27, 28]. Our study applied the CRISPR/Cas9 system to cleave PD-L1 expression in osteosarcoma cells. The position of the PD-L1 sgRNA target is located on exon 2 and 3 of the PD-L1 gene. In this study, the efficiency of sgRNA was first powerfully confirmed *in vitro*. The efficient enrichment of cells enabled the isolation of permanent cell lines expressing the mutations via fluorescent cell sorting. These results demonstrate that PD-L1 sgRNA-guided CRISPR/Cas9 is able to specifically knockout PD-L1 expression. Meanwhile, the establishment of a constitutive PD-L1 knockout cell line offers a good model for the study of osteosarcoma pathology and provides a good basis for the application of CRISPR/Cas9 technology in osteosarcoma treatment. Our data also confirm that the CRISPR/Cas9 system for genome editing is a robust technology that makes it possible to generate cellular models that recapitulate those cooperative alterations rapidly and at low cost.

With the application of CRISPR/Cas9 by PD-L1 sgRNA, we found that PD-L1 was an important factor involved in osteosarcoma cell growth and drug resistance. In order to mimic the *in vivo* environment of osteosarcoma cell growth, 3D cell culture was applied in this study. The diameter of spheroids formed by PD-L1-Cas9 cells was significantly decreased compared with the control cells, which implied that PD-L1 might also be involved in the growth and tumorigenicity of osteosarcoma cells. As migration and invasion capabilities are crucial prerequisites for metastatic osteosarcoma, we compared those functions between PD-L1 knockout cells and control cells. However, there were no significant differences between PD-L1 knockout cells and control cells. Our result showed that migration and invasion of osteosarcoma might not be mediated by PD-L1 alone, but by cell growth or by the PD-1/PD-L1 complex. The function of PD-L1 may be related to the PD-1/PD-L1 complex, which transmits an inhibitory signal to reduce the proliferation of CD8^+^ T cells [20]. With similar results, positive PD-L1 protein expression was considered as an independent predictor for colorectal carcinoma prognosis. Knockdown of PD-L1 can inhibit colorectal carcinoma cell proliferation [29]. Beside metastasis, PD-L1 expression has also been considered as an adequate biomarker to predict responsiveness to these therapies in some cancers. PD-L1 expression in the epithelium or stroma of breast cancer patients predicted complete pathologic response to neoadjuvant chemotherapy in univariate and multivariate analyses [30]. In lymphoma tissue and cell lines, PD-L1 is highly and widely expressed and it may resist the effects of cisplatin on lymphoma cells through anti-apoptotic mechanisms [31]. Consistent with those data, our study showed that PD-L1 played an important role in osteosarcoma cell reaction to chemotherapy. Compared with the control osteosarcoma cells, PD-L1-Cas9 cells showed lower values of IC50 for both doxorubicin and paclitaxel. Taking these data together, we propose that PD-L1 may take part in the drug resistance and tumorigenicity of osteosarcoma, and thereby might be a potential target in clinical treatment.

This study confirms that PD-L1 is highly expressed in osteosarcoma cell lines and tissues. Moreover, this is the first application of CRISPR/Cas9 targeting the PD-L1 gene in osteosarcoma. Furthermore, we have successfully established the PD-L1 CRISPR/Cas9 system and generated osteosarcoma cell lines with constitutive knockout of PD-L1 expression. With the simplicity and flexibility of CRISPR/Cas9, it is expected that the PD-L1 CRISPR/Cas9 system may be a promising therapeutic approach for the treatment of osteosarcoma in the future.

## MATERIALS AND METHODS

### Cell lines and culture

The human osteoblast cells NHOst and HOB-c were purchased from Lonza Walkersville (Walkersville, MD) and PromoCell GmbH (Heidelberg, Germany), respectively. The human osteosarcoma cell lines, U-2OS, SaOS, MG63, MNNG/HOS, and 143B were obtained from the American Type Culture Collection (Rockville, MD). The human osteosarcoma KHOS cell line was kindly provided by Dr. Efstathios Gonos (Institute of Biological Research & Biotechnology, Athens, Greece). The osteosarcoma cell lines were cultured in RPMI-1640 (Life Technologies, Grand Island, NY) supplemented with 10% fetal bovine serum, 100 U/mL penicillin, and 100 mg/mL streptomycin (Life Technologies, Carlsbad, CA). The osteoblast cell lines were cultured in osteoblast growth medium (PromoCell) with 10% fetal bovine serum. All cells were maintained in a humidified atmosphere containing 5% CO_2_, 95% air atmosphere at 37°C.

### Osteosarcoma tissue microarray (TMA) and immunohistochemical staining

Osteosarcoma TMAs were generated as described previously [32, 33]. PD-L1 expression was evaluated by immunohistochemistry as previously described [34]. Primary PD-L1 antibody (1:50 dilution, Abcam, MA) was incubated with the TMA at 4°C overnight in a humidified chamber. Expression of PD-L1 was evaluated according to staining intensities in the cytoplasm of tumor samples. Thereby, staining patterns were categorized into four groups: no staining (0) and weak staining (1+) specimens, which were classified as PD-L1-low patients; and moderate staining (2+) and intense staining (3+), which were classified as PD-L1-high patients. PD-L1 staining images were obtained using a Nikon Eclipse Ti-U fluorescence microscope (Nikon Corp) with a SPOT RT digital camera (Diagnostic Instruments Inc.).

### Western blotting assay

Osteosarcoma and osteoblast cells were lysed with RIPA Lysis Buffer (Upstate Biotechnology, Charlottesville, VA) plus complete protease inhibitor cocktail tablets (Roche Applied Science, IN). Protein concentrations were calculated by the Bio-Rad Protein Assay (Bio-Rad, CA). Antibodies against PD-L1 (1:1000 dilution), Cas9 (1:1000 dilution) were purchased from Abcam, and β-actin (1:2000 dilution) were purchased from Sigma-Aldrich, respectively. Secondary antibodies IRDye^®^ 800CW and IRDye^®^ 680LT were obtained from LI-COR (Biosciences, NE). Western blot analysis was performed as previously reported [35]. Western blot detection and quantitation was performed by the Odyssey infrared imaging system (LI-COR Biosciences, NE).

### CRISPR/Cas9 plasmid design and purification

The CRISPR/Cas9 and green fluorescent protein (GFP) fusion protein expression vector guided by PD-L1 single guide RNA (sgRNA) (abbreviated as PD-L1-Cas9-GFP) was obtained from DNA 2.0 (Newark, CA). GFP is co-expressed from the same mRNA as the Cas9 protein via a NLS peptide linkage, which enables tracking of transfection efficiency. The exon of the PD-L1 gene selected for guiding RNA design is located at the second and third coding exons. The oligo nucleotides used for construction of the PD-L1 gene sgRNA are: 5′AGCAAATATCCTCATCTTTC3′NGG (#1); 5′TCTTTATATTCATGACCTAC3′NGG (#2); 5′ ATTTACTGTCACGGTTCCCA 3′NGG (#3); 5′ TACCATACTCTACCACATAT3′NGG (#4); 5′ AATAGACAATTAGTGCAGCC3′NGG (#5).

### Verification of PD-L1 CRISPR/Cas9 *in vitro*

In order to verify the effectiveness of the PD-L1 sgRNA, we performed the genome editing with “Guide-it technology”. The “Guide-it sgRNA *In Vitro* Transcription and Screening Systems” kit was purchased from Clontech Laboratories Inc. (CA). Firstly, a DNA template that contained the sgRNA encoding sequence under the control of a T7 promoter was generated by performing a PCR with the included Guide-it Scaffold Template and the designed primer. The template was then transcribed with the included Guide-it T7 Polymerase Mix to create a sgRNA containing the target PD-L1 sequence. Subsequently, we purified the sgRNA using digestion with DNAseI, phenol: chloroform extraction, and ethanol precipitation, and measured the concentration. After that, the cleavage template that contained the target sequence in an asymmetric position was expanded by PCR. A cleavage reaction on this template was then performed by using the purified sgRNA in combination with the included Guide-it Recombinant Cas9 Nuclease. Finally, the efficiency of the cleavage reactions was analyzed on an agarose gel to determine if the target sequence had been successfully incorporated.

### Monoclone and PD-L1 verification

The stable osteosarcoma cell lines (KHOS and MNNG/HOS) were transfected with the specific PD-L1-Cas9-GFP vector using Lipofectamine3000 (Invitrogen, Grand Island, NY) according to the manufacturer's instructions, and the cells were allowed to recover for 48 hours before fluorescence activated cell sorting (FACS). Forty-eight hours after transfection, delivery of sgRNA plasmids was confirmed by visualizing green fluorescent marker expression by fluorescence microscopy. Cells dispersed from the culture plate were pelleted and resuspended in 1ml PBS. Using FACS, green fluorescent cells were collected. Individual GFP-positive cells were diluted to 5 cells/ml and the clones in 96-well plates were allowed to expand for seven days. Westernblot was used to identify PD-L1 protein expression and to confirm the cell clones with constitutively knockout PD-L1 expression after FACS. The clones confirmed with constitutive knockout of PD-L1 expression were used for the following functional assays.

### Drug resistance evaluation by MTT cell proliferation assay

Doxorubicin and paclitaxel, which are the most common chemotherapy choices for osteosarcoma treatment, were provided by the pharmacy at the Massachusetts General Hospital Cancer Center. The stock solutions of these drugs were prepared according to the manufacturers’ specifications and stored at −20°C. Effects of PD-L1 on osteosarcoma drug resistance to doxorubicin and paclitaxel were assessed by the MTT assay. In 96-well plates, 5 × 10^3^ cells/mL (including KHOS, KHOS PD-L1-Cas9, MNNG/HOS, or MNNG/HOS PD-L1-Cas9 cells) were seeded into each well. Complete growth medium without antibiotics was added into each well to a volume of 100 μL in triplicate. Osteosarcoma cells were dosed with doxorubicin in increasing concentration up to10 μM or paclitaxel in increasing concentrations up to 1 μM. After 96 hours of culture, 20 μL of MTT (5 mg/mL) was added to each well and the cells were incubated for 4 hours at 37°C. Absorbance at a wave length of 490 nm was measured on a SPECTR Amax Microplate Spectrophotometer from Molecular Devices (Sunnyvale, CA). All results were analyzed by GraphPad Prism 5 software (San Diego, CA).

### Three dimension culture

A 3D cell culture is an improved method that can simulate the cell growth environment *in vivo* and is a more accurate model to determine the behavior of cancer cell growth. Following the manufacturer's protocol, osteosarcoma cell spheroid formation was established in HDP1096 Perfecta3D^®^ 96-Well Hanging Drop Plates (3D Biomatrix). Hanging drops were formed by pipetting 40 μL of cell suspension (1 × 10^5^/mL) into each well. Medium was changed every other day to provide enough nutrients for cells and to prevent osmolality shift of the medium. After seven days culture, the spheroids were harvested from the bottom side of the plate by gently pipetting 100 μL PBS into each well. After 15 minutes of incubation with 1 μM Calcein AM (Life Technologies), the spheroids were imaged on a Nikon Eclipse Ti-U inverted fluorescence microscope (Nikon Instruments, Inc NY, CA). The size of spheroids was calculated using ImageJ.

### Matrigel invasion assay

Matrigel invasion assay was applied to evaluate the role of PD-L1 in the process of osteosarcoma metastasis. BioCoat^™^ Matrigel^™^ Invasion Chambers (Becton-Dickinson, MA) were used according to the manufacturer's instructions. Osteosarcoma cells (5 × 10^4^ cells/plate) were planted into the upper chamber of each well in serum-free medium, and complete medium was put into the bottom chamber. After incubation for 24 hours, non-invaded cells were removed by scrubbing from the upper surface of the membrane with cotton-tipped swabs. After fixation in 100% methanol and staining with hematoxylin, the invaded cells were counted in three images of each membrane under a microscope using a 200× objective.

### Wound healing migration assay

Migration activity is another method to identify the potential role of PD-L1 in the metastasis of osteosarcoma. In this study, migration activity was detected by the multiple scratch wound assay. Osteosarcoma cells (1 × 10^5^ cells/well) were seeded into 12-well plates. Three parallel lines were made in confluent cell cultures with a 200 μL tip. The wounds were observed at 0, 8, and 24 hours after scratching separately, and photographed at each time point using a 10× objective. The cell migration distance was defined as the wound width at the 0 h time point minus the wound width at each time point and then divided by two. The distance between the two edges of the wound width was randomly quantified at 10 sites in each image.

### Statistical analysis

GraphPad Prism5 software (GraphPad Software, Inc.) was used for statistical analyses. Results are expressed as mean ± SD and *P* values < 0.05 were regarded as statistically significant. We used the χ^2^ test to analyze the relationship between PD-L1 expression and clinic pathological parameters of osteosarcoma. The log-rank test was used to compare the differences in survival curves. Prognostic factors associated with overall survival or five-year survival were investigated according to the Cox proportional hazards regression model, in a stepwise manner. Only those factors that were statistically significant (*P* < 0.05) in the univariate survival analysis were included in the multivariate analyses.

## SUPPLEMENTARY MATERIALS AND TABLE


