# Correction: Targeting programmed cell death ligand 1 by CRISPR/Cas9 in osteosarcoma cells

**DOI:** 10.18632/oncotarget.28536

**Published:** 2024-02-05

**Authors:** Yunfei Liao, Lulu Chen, Yong Feng, Jacson Shen, Yan Gao, Gregory Cote, Edwin Choy, David Harmon, Henry Mankin, Francis Hornicek, Zhenfeng Duan

**Affiliations:** ^1^Department of Endocrinology, Wuhan Union Hospital, Tongji Medical College, Huazhong University of Science and Technology, Wuhan 430022, China; ^2^Sarcoma Biology Laboratory, Department of Orthopaedic Surgery, Massachusetts General Hospital and Harvard Medical School, Boston 02114, Massachusetts, USA; ^3^Department of Orthopaedic Surgery, Wuhan Union Hospital, Tongji Medical College, Huazhong University of Science and Technology, Wuhan 430022, China; ^4^Division of Hematology and Oncology, Massachusetts General Hospital and Harvard Medical School, Boston 02114, Massachusetts, USA

**This article has been corrected:** In Figure 1B, the β-actin panel is an accidental duplicate of the β-actin panel in Figure 2D. The corrected Figure 1, produced using the original data, is shown below. The authors declare that these corrections do not change the results or conclusions of this paper.

Original article: Oncotarget. 2017; 8:30276–30287. 30276-30287. https://doi.org/10.18632/oncotarget.16326

**Figure 1:**
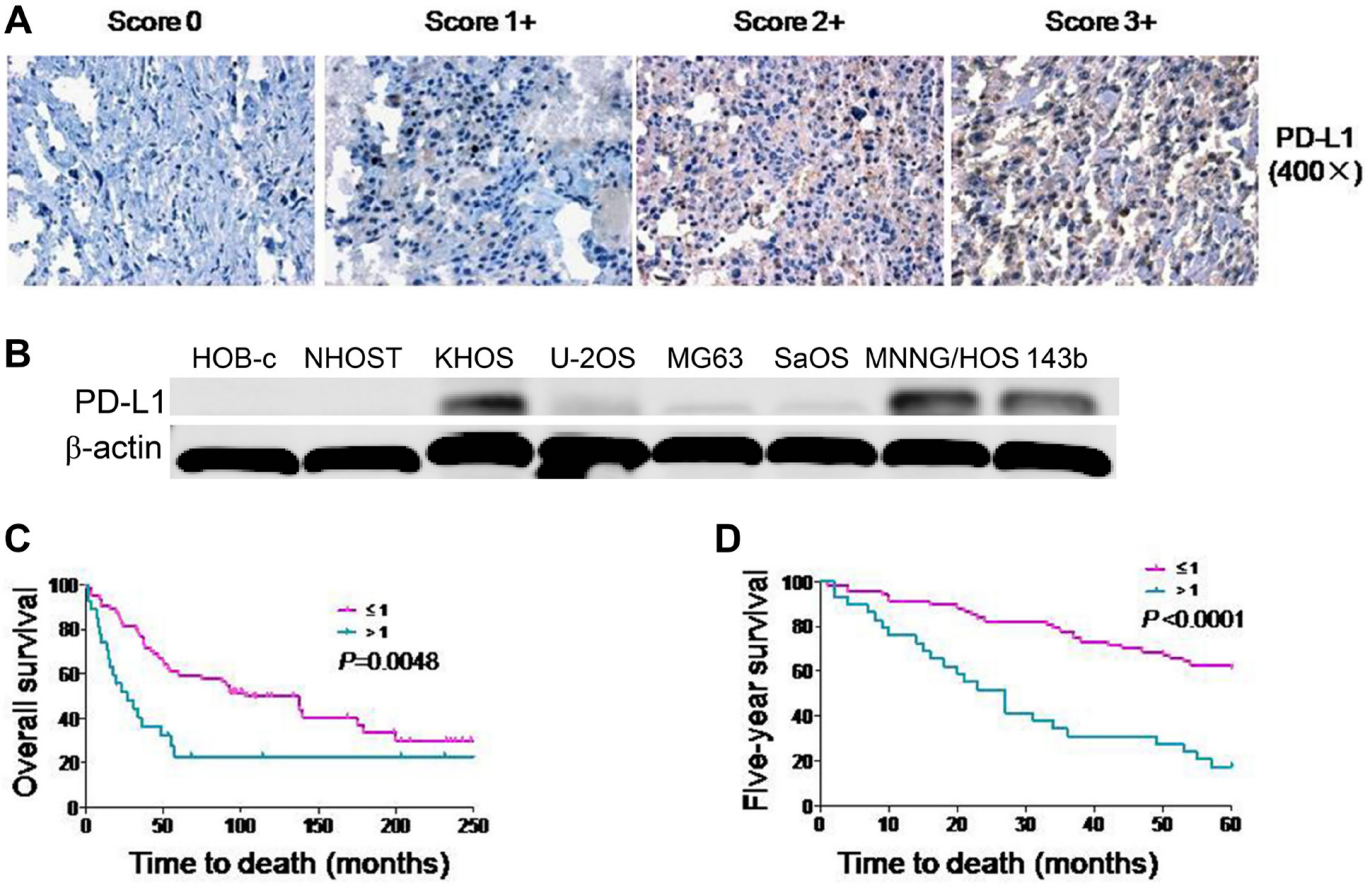
Expression of PD-L1 protein in osteosarcoma cell lines and osteosarcoma patient tissues. (**A**) Representative images of different immunohistochemical staining intensities of PD-L1 are shown in osteosarcoma tissues. The percentage of cells showing positive cytoplasmic staining for PD-L1 was calculated by reviewing the entire spot. Based on the PD-L1 staining intensities in the tumor samples, the staining patterns were categorized into 4 groups: no staining (0), weak staining (1+), moderate staining (2+) and intense staining (3+) (Original magnification, 400×). (**B**) Expression of PD-L1 protein in osteosarcoma cell lines and normal osteoblast cell lines. (**C**) Kaplan-Meier overall survival curve of patients with osteosarcoma were subgrouped as either PD-L1 low staining (staining ≤ 1) or high staining (staining ≥ 2). (**D**) Kaplan-Meier five-year survival curve of patients with osteosarcoma were subgrouped as either PD-L1 low staining (staining ≤ 1) or high staining (staining ≥ 2).

