# Supplementary material for: Targeting programmed cell death ligand 1 by CRISPR/Cas9 in osteosarcoma cells
Source: Oncotarget. 2017 Mar 17;8(18):30276–87. doi: 10.18632/oncotarget.16326 (PMC5444742; doi:10.18632/oncotarget.16326)
Supplement: Supplementary file 1 [file oncotarget-08-30276-s001.pdf]

Targeting programmed cell death ligand 1 by CRISPR/Cas9 in osteosarcoma cells

Supplementary Materials

Supplementary Table 1: Multivariate survival analysis

Variable	Overall survival			Five-year survival		
	**Hazard Ratio**	**95% CI**	***P***	**Hazard Ratio**	**95% CI**	***P***
Metastasis	3.910	1.937–6.279	< 0.001	4.352	1.976–6.891	0.003
PD-L1 expression	2.114	1.485–3.233	0.045	3.259	2.078–5.185	0.009
